# Pathological roles of ubiquitination and deubiquitination systems in sepsis-induced myocardial dysfunction

**DOI:** 10.17305/bb.2024.11738

**Published:** 2025-01-07

**Authors:** Zhiping Wang, Simiao Sun, Lili Huang, Xinlong Chen, Huifen Xu, Hongwei Ma, Mingbing Xiao, Linhua Wang

**Affiliations:** 1 Department of Critical Care Medicine, Affiliated Hospital of Nantong University, Medical school of Nantong University, Jiangsu, China; 2 Nantong Fourth People’s Hospital, Jiangsu, China; 3 Department of Gastroenterology, Affiliated Hospital of Nantong University, Medical School of Nantong University, Jiangsu, China; 4 Department of Laboratory Medicine, Affiliated Hospital and Medical School of Nantong University, Jiangsu, China

**Keywords:** Sepsis-induced myocardial dysfunction, SIMD, ubiquitination, deubiquitination, E3 ligases, deubiquitinating enzymes, DUBs

## Abstract

Sepsis-induced myocardial dysfunction (SIMD) is a severe complication of sepsis, characterized by impaired cardiac function and high mortality rates. Despite significant advances in understanding sepsis pathophysiology, the molecular mechanisms underlying SIMD remain incompletely elucidated. Ubiquitination and deubiquitination, critical post-translational modifications (PTMs) regulating protein stability, localization, and activity, play pivotal roles in cellular processes, such as inflammation, apoptosis, mitochondrial function, and calcium handling. Dysregulation of these systems has been increasingly implicated in the pathogenesis of SIMD. This review provides a comprehensive overview of the pathological mechanisms driving SIMD, with a focus on the classification and functions of E3 ubiquitin ligases and deubiquitinating enzymes (DUBs), their regulatory systems, and their involvement in SIMD. Dysfunction of the ubiquitin-proteasome system (UPS), often driven by altered activity of E3 ligases, accelerates the degradation of critical regulatory proteins, thereby exacerbating cardiac inflammation, oxidative stress, and apoptosis. Concurrently, imbalances in DUB activity disrupt protein homeostasis, further amplifying myocardial injury. Emerging research underscores the therapeutic potential of targeting these systems. Strategies aimed at modulating E3 ligase activity or restoring DUB balance have shown promise in preclinical studies. This review summarizes current findings on the roles of ubiquitination and deubiquitination in SIMD pathogenesis, highlights the key challenges in advancing this field, and proposes directions for future research.

## Introduction

**Figure 1.**
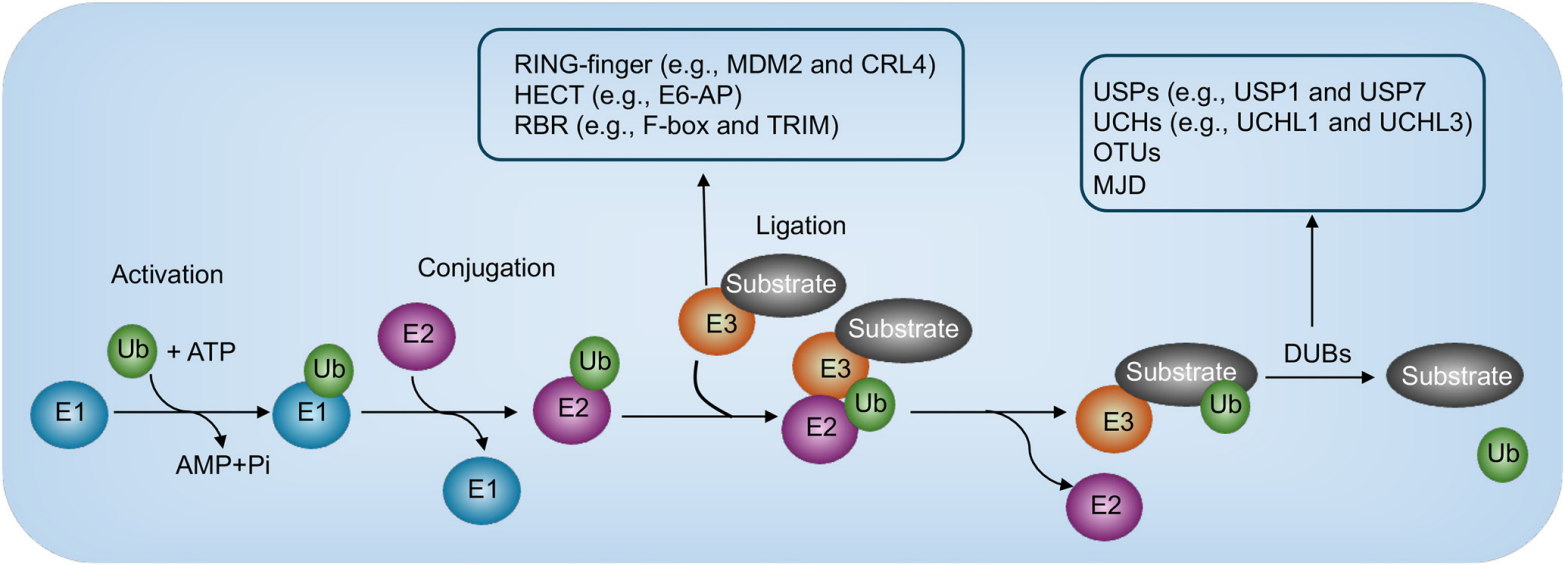
**Overview of key steps in ubiquitination and deubiquitination.** The ubiquitination process begins with the activation of ubiquitin by the E1-activating enzyme in an ATP-dependent reaction. Activated ubiquitin is subsequently transferred to an E2-conjugating enzyme. The E3 ligase facilitates the attachment of ubiquitin to a substrate. Deubiquitinating enzymes reverse this modification by removing ubiquitin from the substrates.

Sepsis, a life-threatening condition triggered by a dysregulated host response to infection, remains one of the leading causes of mortality among critically ill patients [1, 2]. Among the organ systems affected, the cardiovascular system is particularly vulnerable, often resulting in sepsis-induced myocardial dysfunction (SIMD) [3, 4]. SIMD is characterized by reversible cardiac impairments, including reduced myocardial contractility, impaired ventricular ejection, and bioenergetic deficits [3, 4]. Despite its recognition as a key determinant of sepsis outcomes, the molecular mechanisms driving SIMD remain poorly understood, which complicates the development of effective therapies. Post-translational modifications (PTMs) play a crucial role in regulating cellular responses to stress, infection, and injury [5, 6]. Ubiquitination and deubiquitination, which involve the addition and removal of ubiquitin moieties on target proteins, are highly dynamic PTMs that influence protein stability, localization, and activity (Figure 1) [5, 6]. The ubiquitin-proteasome system (UPS) and deubiquitinating enzymes (DUBs) have been shown to regulate critical processes, such as inflammation, apoptosis, oxidative stress, and mitochondrial function—all of which contribute to SIMD [3, 4]. Dysregulation of these systems has been implicated not only in various cardiovascular diseases but also increasingly in SIMD. Recent studies have identified key components of the ubiquitination and deubiquitination systems that are involved in the pathogenesis of SIMD. For example, altered activity of E3 ubiquitin ligases and imbalances in DUBs have been associated with enhanced protein degradation, mitochondrial dysfunction, and apoptotic signaling in cardiac tissue [7–13]. This review provides a comprehensive overview of the pathological roles of the ubiquitination and deubiquitination systems in SIMD. It discusses the mechanisms through which these systems contribute to myocardial injury and explores their potential as therapeutic targets. By highlighting recent advances and identifying knowledge gaps, we aim to underscore the importance of these regulatory pathways in the context of sepsis and cardiac dysfunction.

## Molecular mechanisms of SIMD

SIMD is a multifaceted condition driven by complex molecular pathways that disrupt normal cardiac function [3, 4]. Key mechanisms involved include inflammation, oxidative stress, mitochondrial dysfunction, and apoptotic signaling [3, 4]. Collectively, these processes contribute to myocardial damage, bioenergetic failure, and contractile dysfunction [3, 4]. This section delves into the molecular underpinnings of SIMD, highlighting how these interconnected pathways drive disease progression.

### Inflammatory responses and cytokine storm

**Figure 2.**
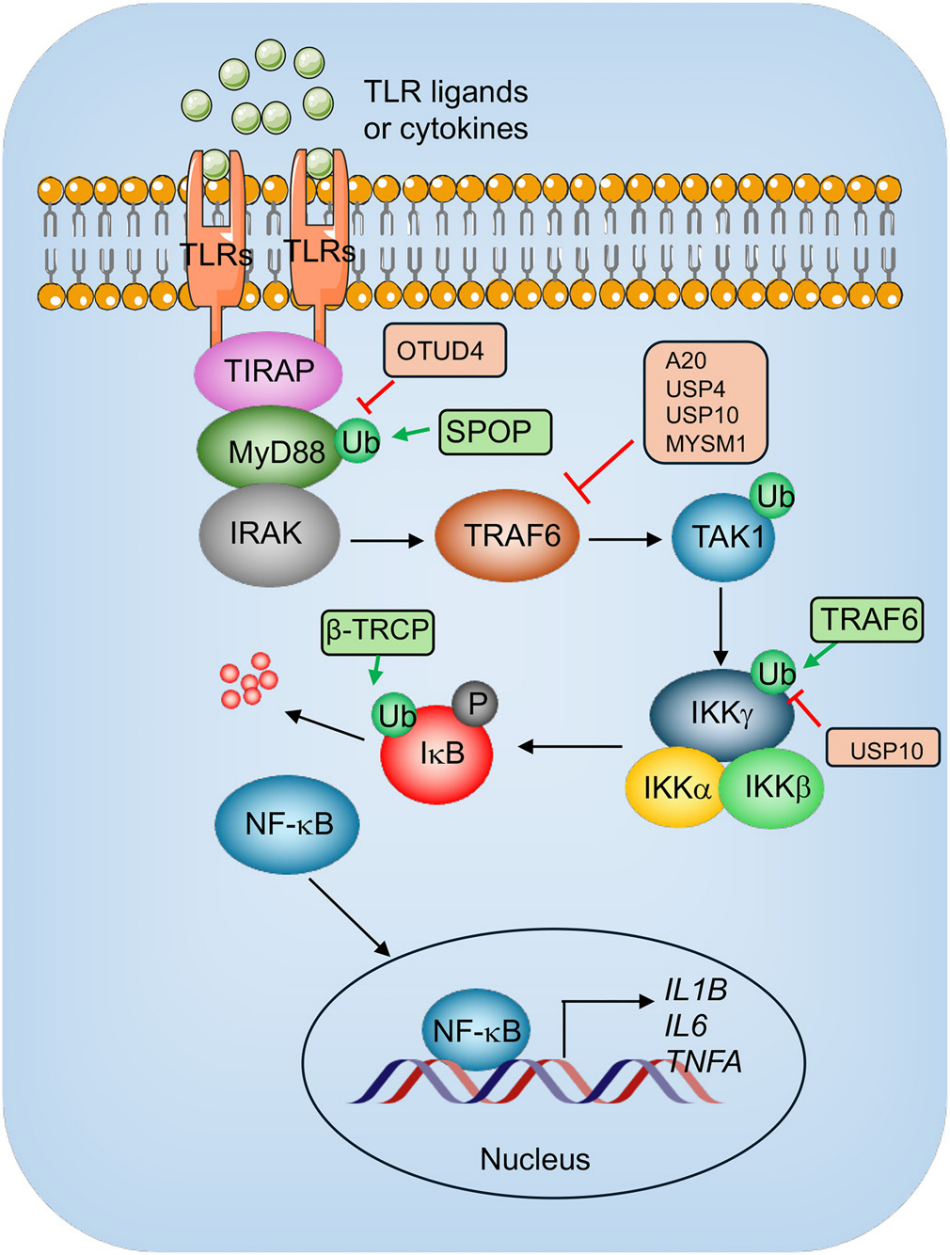
**TLR/NF-κB mediated inflammatory signaling and involvement of E3 and DUBs.** Upon the activation of TLRs by their respective ligands or cytokines, TLR2/4 and TLR7/8/9 recruit adaptor proteins, such as TIRAP, MyD88, and RAKs, leading to the assembly of the MyD88 signaling complex. TRAF6 is subsequently engaged, catalyzing the synthesis of K63-linked polyubiquitin chains that serve as scaffolds for recruiting TAK1 and the IKK complex (IKKα, IKKβ, and IKKγ). The IKK complex phosphorylates IκB, marking it for ubiquitination and degradation. This process releases NF-κB, composed of p50 and p65 subunits, allowing its translocation into the nucleus to promote the transcription of pro-inflammatory genes, such as IL-1β, IL-6, and TNF-α. E3 ligases, represented by green rectangles, ubiquitinate specific components in the pathway, while DUBs, depicted as orange rectangles, remove ubiquitin modifications to regulate signaling. TLR: Toll-like receptor; NF-κB: Nuclear factor-kappa B; DUB: Deubiquitinating enzyme; IL-1β: Interleukin-1 beta; IL-6: Interleukin-6; TNF-α: Tumor necrosis factor-alpha; MYD88: Myeloid differentiation primary response 88; IKK: IκB kinase; IKKγ: Inhibitor of NF-κB kinase subunit gamma.

The hyperactivation of the immune system during sepsis leads to a cytokine storm, characterized by the excessive release of pro-inflammatory cytokines, including tumor necrosis factor-alpha (TNF-α), interleukin-1 beta (IL-1β), and interleukin-6 (IL-6) [1, 2]. These cytokines contribute to cardiac depression by impairing cardiomyocyte contractility, disrupting calcium handling, and promoting endothelial dysfunction [1, 2]. Inflammation is regulated by a complex interplay of molecular and cellular mechanisms that maintain homeostasis while responding to injury or infection [14, 15]. Toll-like receptor (TLR) signaling plays a critical role in initiating inflammatory responses, primarily through the activation of nuclear factor-kappa B (NF-κB) (Figure 2) [14, 15]. Upon recognizing pathogen-associated molecular patterns (PAMPs), TLRs recruit adaptor proteins such as myeloid differentiation primary response 88 (MyD88) or TIR-domain-containing adapter-inducing interferon-β (TRIF). MyD88-dependent signaling involves the recruitment of IL-1 receptor-associated kinases (IRAKs) and TNF receptor-associated factor 6 (TRAF6), which catalyzes K63-linked ubiquitination to activate transforming growth factor-β-activated kinase 1 (TAK1) [14, 15]. TAK1 phosphorylates the IκB kinase (IKK) complex, triggering the degradation of IκB and releasing NF-κB for nuclear translocation (Figure 2) [14, 15]. Activated NF-κB drives the production of pro-inflammatory cytokines (e.g., TNF-α, IL-1, and IL-6) and chemokines, which recruit immune cells to the site of injury [14, 15]. PTMs, such as ubiquitination and deubiquitination, fine-tune TLR-NF-κB signaling networks, ensuring an appropriate inflammatory response [14, 15]. Resolution of inflammation involves processes like efferocytosis, which clears apoptotic cells, and the production of anti-inflammatory mediators, ultimately restoring tissue integrity and function [14, 15]. Dysregulation of these mechanisms, including overactivation of TLR pathways, can result in chronic inflammation and related diseases [14, 15].

### Oxidative stress and reactive oxygen species (ROS)

Oxidative stress and ROS are key components of cellular signaling and damage in various pathological conditions, including SIMD [16, 17]. ROS are highly reactive molecules produced as byproducts of normal cellular metabolism, particularly in mitochondria during aerobic respiration [16, 17]. While these molecules are essential for processes, such as gene expression, immune responses, and tissue repair, excessive ROS production—often triggered by metabolic and inflammatory stress—can cause oxidative damage to lipids, proteins, and DNA, ultimately leading to cellular dysfunction and injury [16, 17]. In SIMD, oxidative stress is significantly elevated due to the intensified inflammatory response and mitochondrial dysfunction associated with sepsis [18–20]. The activation of immune cells, such as neutrophils and macrophages, during sepsis generates large quantities of ROS, overwhelming cellular antioxidant defenses [18–20]. This oxidative stress disrupts mitochondrial function, impairs calcium handling, and promotes both apoptotic and necrotic cell death, thereby exacerbating myocardial injury [18–20]. Dysregulated antioxidant enzyme activity and the imbalance between ROS production and clearance are central to the pathophysiology of SIMD [18–20]. Targeting oxidative pathways and restoring redox balance through therapeutic interventions offer a promising strategy to reduce myocardial damage and improve cardiac function in sepsis.

### Mitochondrial dysfunction

Mitochondria play a central role in cellular energy production and homeostasis [21]. In SIMD, mitochondrial dysfunction manifests through impaired oxidative phosphorylation, reduced ATP production, and increased ROS generation [17–20]. Additionally, the loss of mitochondrial membrane potential and the release of cytochrome c into the cytosol exacerbate apoptotic signaling pathways [22]. This dysfunction is often associated with disrupted calcium homeostasis, oxidative damage, and altered mitochondrial dynamics, characterized by increased fission and decreased fusion [17–20].

### Apoptosis and cardiomyocyte death

**Figure 3.**
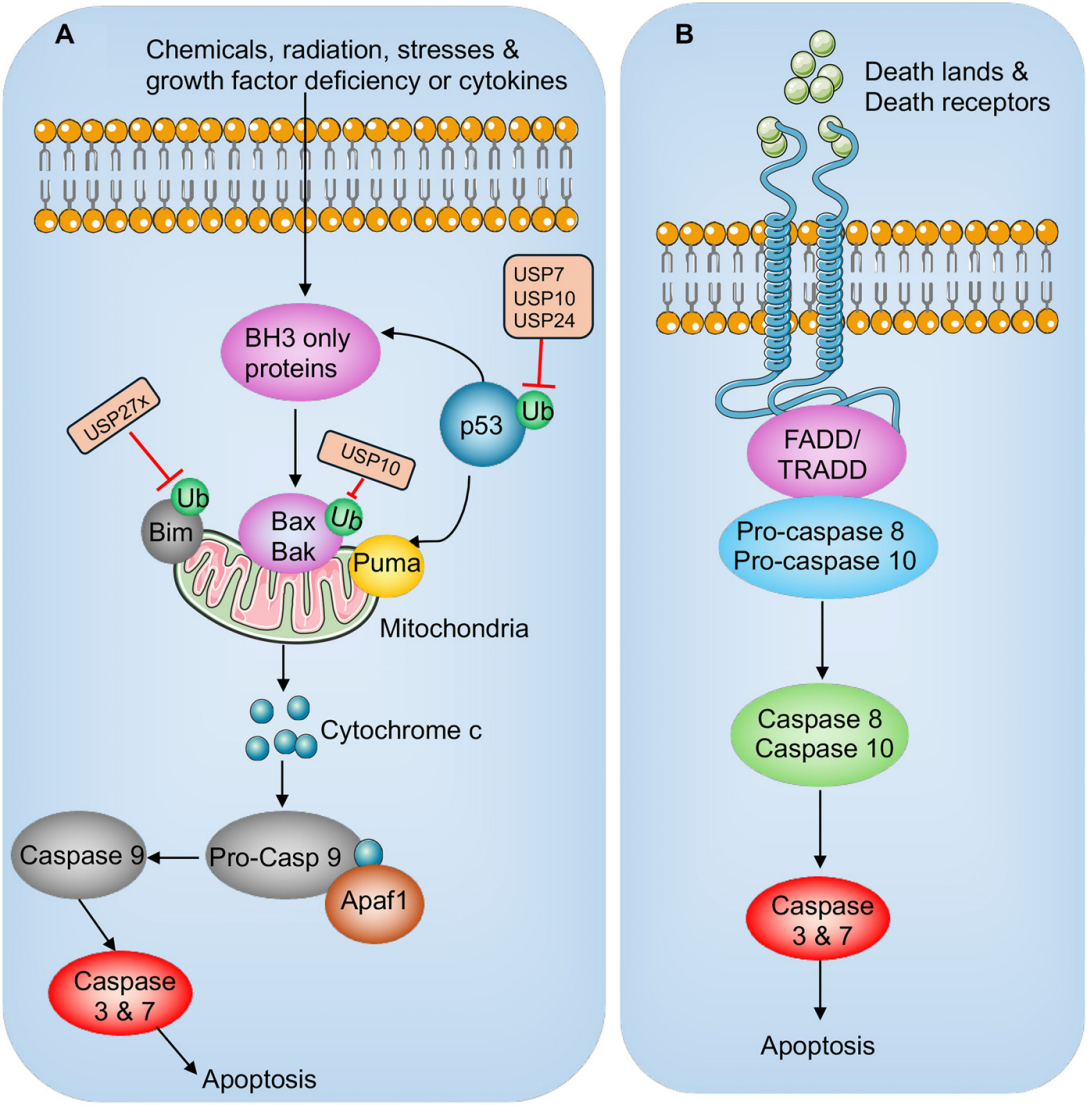
**Extrinsic and intrinsic apoptosis signaling pathway and involvement of E3 and DUBs.** (A) Intrinsic apoptosis. Triggered by stressors like DNA damage, ER stress, or growth factor deprivation, intrinsic apoptosis involves activation of BH-3-only proteins, which promote pro-apoptotic factors (e.g., p53, Bax, Bak). This causes mitochondrial outer membrane permeabilization and cytochrome c release. Cytochrome c forms the apoptosome with Apaf1 and procaspase-9, leading to caspase-9 activation. Caspase-9 then activates effector caspases (caspase-3/7), executing apoptosis. (B) Extrinsic apoptosis. The extrinsic pathway begins with death receptor activation, recruiting FADD and TRADD to form the DISC. Procaspase-8 is activated at the DISC, regulated by c-FLIP. Activated caspase-8 then cleaves and activates effector caspases (caspase-3/7), driving apoptosis. E3 ligases, represented by green rectangles, ubiquitinate specific components in the pathway, while DUBs, depicted as orange rectangles, remove ubiquitin modifications to regulate signaling. DUB: Deubiquitinating enzyme; DISC: Death-inducing signaling complex; FADD: Fas-associated death domain.

Apoptosis, or programmed cell death, is a critical driver of cardiomyocyte loss in SIMD [3, 4, 23–25]. During sepsis, both the extrinsic (death receptor-mediated) and intrinsic (mitochondria-mediated) apoptotic pathways are activated [3, 4, 23–25]. The intrinsic pathway is initiated by intracellular stress signals, such as DNA damage or oxidative stress, and is regulated by the B-Cell CLL/Lymphoma 2 (BCL-2) protein family. Pro-apoptotic members of this family, including BCL2-associated X (Bax) and BCL2 antagonist/killer 1 (Bak), promote mitochondrial outer membrane permeabilization (MOMP), leading to the release of cytochrome c into the cytosol [23–25]. Once in the cytosol, cytochrome c binds to apoptotic peptidase activating factor 1 (Apaf1), forming the apoptosome. This complex activates caspase-9, which in turn triggers downstream executioner caspases, such as caspase-3 and caspase-7 (Figure 3A) [23–25]. The extrinsic pathway, on the other hand, is triggered by ligand binding to death receptors, such as fas or TNF receptors, on the cell surface [23–25]. This ligand–receptor interaction recruits adaptor proteins like fas-associated death domain (FADD) to form the death-inducing signaling complex (DISC), which activates caspase-8 (Figure 3B) [23–25]. Activated caspase-8 can either directly activate executioner caspases or cleave BH3-interacting domain death agonist (BID), effectively linking the extrinsic and intrinsic pathways and amplifying mitochondrial signaling [23–25]. Both apoptotic pathways are tightly regulated by specific proteins to ensure proper and controlled execution of cell death processes.

### Impaired calcium handling and contractility

**Figure 4.**
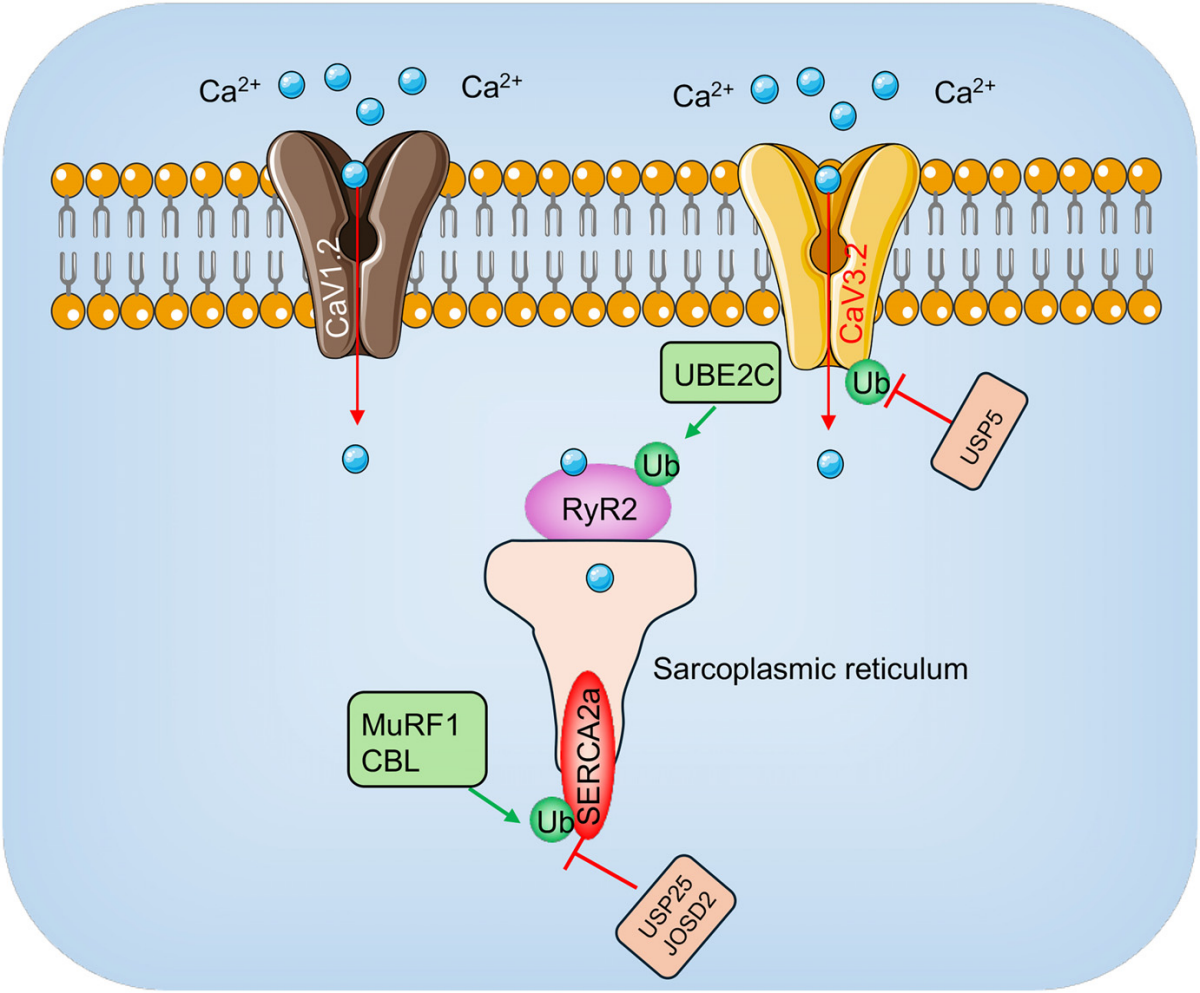
**Involvement of E3 ligases and DUBs in calcium handling.** The influx of Ca^2+^ through voltage-gated Ca^2+^ channels, such as CaV1.2 and CaV3.2, initiates a cascade of events. The inward Ca^2+^ current activates RyR2 channels, resulting in the coordinated release of sarcoplasmic reticulum Ca^2+^. This SR-released Ca^2+^ contributes significantly to the rise in intracellular Ca^2+^ concentration that activates myofilaments, facilitating the formation of actin-myosin cross-bridges and driving myocardial contraction. E3 ligases, represented by green rectangles, ubiquitinate specific components in the pathway, while DUBs, depicted as orange rectangles, remove ubiquitin modifications to regulate signaling. DUB: Deubiquitinating enzyme.

Calcium handling is crucial for myocardial contraction and relaxation, as it regulates excitation–contraction coupling in cardiomyocytes [26, 27]. The sarcoplasmic/endoplasmic reticulum calcium ATPase 2a (SERCA2a) plays a pivotal role in this process by pumping calcium ions back into the sarcoplasmic reticulum (SR) after contraction, thereby facilitating muscle relaxation (Figure 4) [28]. In addition, calcium influx through voltage-gated calcium channels (VGCCs) and ryanodine receptors (RyR2) is vital for initiating contraction, while calcium efflux is controlled by the sodium–calcium exchanger (NCX) [28]. Proper regulation of these mechanisms ensures coordinated heart function and rhythmic contractions. In SIMD, disruptions in calcium handling are major contributors to cardiac dysfunction. Impaired SERCA2a activity—caused by reduced expression or functional alterations—leads to inefficient calcium reuptake, which prolongs contraction and hinders proper relaxation [29–31]. This results in diminished cardiac performance. Furthermore, oxidative stress and elevated ROS in sepsis worsen these calcium handling defects by modifying calcium channels and associated proteins [32].

### Crosstalk among pathways

The molecular mechanisms underlying SIMD are deeply interconnected rather than isolated. For instance, oxidative stress can intensify inflammatory responses, while mitochondrial dysfunction worsens both oxidative stress and apoptotic signaling [3, 4]. Similarly, impaired calcium handling not only reduces contractility but also triggers mitochondrial dysfunction and promotes cardiomyocyte death [3, 4]. These interconnections emphasize the complexity of SIMD and the pressing need for comprehensive, multifaceted therapeutic strategies.

## Ubiquitination and deubiquitination process

### Ubiquitination process

The ubiquitination process involves the covalent attachment of a small protein called ubiquitin to target proteins. This modification can signal for their degradation by the proteasome, alter their cellular localization, or influence their interactions with other cellular components [33]. Ubiquitination is facilitated by a complex enzymatic cascade that includes three key enzymes: E1 (ubiquitin-activating enzyme), E2 (ubiquitin-conjugating enzyme), and E3 (ubiquitin ligase) (Figure 1) [6, 7, 33]. The cascade begins with the E1 enzyme, which activates ubiquitin by forming a thioester bond with it in an ATP-dependent reaction [6, 7, 33]. While there is only one E1 enzyme in humans, it is capable of activating ubiquitin for a wide range of substrates [6, 7, 33]. Next, the E2 enzymes transfer the activated ubiquitin from the E1 enzyme to the substrate protein [6, 7, 33]. Humans have over 30 E2 enzymes, each exhibiting specificity for certain types of ubiquitination. Finally, E3 ligases play a critical role in substrate recognition, as they confer specificity by identifying particular target proteins and catalyzing the transfer of ubiquitin onto them [6, 7, 33]. Ubiquitination can involve either the addition of a single ubiquitin molecule (monoubiquitination) or the formation of a chain of ubiquitin molecules (polyubiquitination). Different types of polyubiquitin chains often serve as distinct signals, directing specific cellular outcomes such as proteasomal degradation or endocytosis [6, 7, 33].

### Classes and functions of E3 ligases

E3 ligases are categorized into several families based on their structural domains and mechanisms of action. The largest and most diverse group is the really interesting new gene (RING) E3 ligases, which contain a RING domain that coordinates zinc ions to facilitate ubiquitin transfer from the E2 conjugating enzyme to the target protein [6, 7, 33]. These ligases act as scaffolds, bringing the E2 enzyme and substrate into close proximity without undergoing conformational changes [6, 7, 33]. Notable examples include mouse double minute 2 homolog (MDM2) and the Cullin 4-RING E3 ligase (CRL4) family. MDM2 regulates the tumor suppressor p53, while casitas B lineage lymphoma (c-CBL) mediates receptor endocytosis and signal transduction [34]. The CRL4 ligase complex consists of the Cullin 4 (Cul4) scaffold protein, RING-box protein 1 (Rbx1 or Rbx2), and a substrate receptor such as damaged DNA-binding protein 1 (DDB1) or other specialized receptor proteins [35, 36]. CRL4 ligases play a central role in cellular homeostasis by targeting key regulatory proteins for degradation, including cyclins, CDK inhibitors, tumor suppressors, and transcriptional repressors [35, 36]. The second category, homologous to E6-AP carboxyl terminus (HECT) E3 ligases, undergoes a conformational change to form a thioester bond with ubiquitin before transferring it to the substrate [37]. These ligases are involved in apoptosis, cell signaling, and DNA damage repair [37]. Key examples include E6-associated protein (E6-AP), which facilitates the degradation of tumor suppressor proteins, and neural precursor cell expressed developmentally down-regulated protein 4 (NEDD4), which regulates membrane protein activity [37]. The third category, RING-between-RING (RBR) E3 ligases, features two RING domains separated by a linker region and plays a role in processes like mitophagy [38]. A well-studied example is Parkin, which regulates mitochondrial quality control by targeting damaged mitochondria for degradation [39]. Other types of E3 ligases, such as F-box E3 ligases and Tripartite Motif (TRIM) E3 ligases, further expand this enzyme family’s diversity. These ligases contribute to various cellular processes, including cell cycle regulation and immune responses [40, 41]. The primary function of E3 ligases is to confer substrate specificity, targeting proteins for proteasomal degradation or regulating their function through other mechanisms. This regulation affects protein levels in key processes, such as signaling pathways, DNA repair, immune responses, and cell cycle control [36–40].

### DUBs and deubiquitination process

Deubiquitination is the process by which ubiquitin is removed from substrates, often reversing the effects of ubiquitination (Figure 1) [7]. This process is mediated by a group of enzymes known as DUBs [7]. DUBs are critical for maintaining the balance between ubiquitin conjugation and removal, thereby modulating protein stability, activity, and function [7]. They are classified into two major families: ubiquitin-specific proteases (USPs) and ubiquitin C-terminal hydrolases (UCHs) [7]. USPs, such as USP1 and USP7, represent the largest DUB family and are involved in processes like DNA repair and cell cycle regulation [42]. UCHs, including UCHL1 and UCHL3, primarily cleave ubiquitin chains from substrates, playing an essential role in protein homeostasis [43]. Other DUB families, such as ovarian tumor domain-containing proteases (OTUs) and Machado–Joseph disease (MJD) proteins, regulate diverse signaling pathways, including immune responses and protein quality control [44]. Beyond these, DUBs contribute to cellular processes like signal transduction, stress adaptation, and immune regulation [7]. The dynamic interplay between ubiquitination and deubiquitination ensures precise control of protein levels and functions, while disruptions in this balance are linked to the pathogenesis of various diseases, including cancer, neurodegenerative disorders, and SIMD [44].

## Ubiquitination in SIMD

To date, numerous published studies have demonstrated that a variety of E3 ubiquitin ligases play critical roles in regulating biological processes, such as inflammation, mitochondrial function, and apoptosis. These processes, in turn, mediate the development and progression of SIMD (Table 1).

**Table 1:** E3 ligases involved in various biological processes in the pathogenesis of SIMD

**E3 ligases**	**Substrates**	**Biological processes**	**References**
TRAF6	IKKγ TAK1	Inflammation	[45, 46]
β-TRCP	IκBα	Inflammation	[47]
SPOP	MyD88	Inflammation	[48]
TRAF6 cIAP1 cIAP2 XIAP	RIPK2	Inflammation	[53]
CRL4^DCAF8^	NcoR1	Inflammation	[54]
Parkin	VDAC1 MFN2	Mitochondrial dysfunction	[58, 59]
AMFR	MFN1 MFN2	Mitochondrial dysfunction	[60, 61]
MARCH5	DRP1 MFN1 MFN2 SOD1	Mitochondrial dysfunction	[62–64]
Mul1	MFN2	Mitochondrial dysfunction	[66]
TRIM31	TAK1	Apoptosis	[69]
cIAP1 cIAP2	RIP1	Apoptosis	[70]
TRAF6	Akt2	Apoptosis	[11]
MuRF1	SERCA2a	Calcium handling	[72]
CBL	SERCA2a	Calcium handling	[74]

SIMD: Sepsis-induced myocardial dysfunction; TRAF6: TNF receptor-associated factor 6; IKKγ: Inhibitor of NF-κB kinase subunit gamma; TAK1: Transforming growth factor-β-activated kinase 1; β-TRCP: Beta-transducin repeat-containing E3 ubiquitin protein ligase; IκBα: NF-κB inhibitor alpha; SPOP: Speckle-type BTB-POZ protein; MYD88: Myeloid differentiation primary response 88; RIPK2: Receptor-interacting serine–threonine kinase 2; cIAP1: Cellular inhibitor of apoptosis 1; cIAP2: Cellular inhibitor of apoptosis 2; XIAP: X-linked inhibitor of apoptosis; CRL4: Cullin 4-RING E3 ligase; NcoR1: Nuclear receptor corepressor 1; VDAC1: Voltage-dependent anion channel 1; MFN2: Mitofusin-2; MFN1: Mitofusin-1; AMFR: Autocrine motility factor receptor; MARCH5: Membrane-associated RING-CH finger protein 5; Mul1: Mitochondrial ubiquitin ligase 1; DRP1: Dynamin-related protein 1; SOD1: Superoxide dismutase 1; RIP1: Receptor-interacting protein 1; MuRF1: Muscle RING finger-1; CBL: Casitas B-lineage lymphoma; SERCA2a: Sarcoplasmic/endoplasmic reticulum calcium ATPase 2a.

### Ubiquitination and inflammatory signaling

In sepsis, ubiquitination plays a critical role in regulating inflammatory pathways [8, 9]. Activation of TLRs by bacterial components triggers downstream signaling cascades, many of which are tightly controlled by ubiquitination (Figure 2). For instance, the RING-type E3 ligase TRAF6 mediates K63-linked ubiquitination of key signaling proteins, such as inhibitor of NF-κB kinase subunit gamma (IKKγ) and TAK1 [45, 46], thereby promoting the activation of NF-κB. Another important regulator, beta-transducin repeat-containing E3 ubiquitin protein ligase (β-TRCP), a member of the SKP1–Cullin–F-box (SCF) E3 ligase family, specifically recognizes phosphorylated destruction motifs in NF-κB inhibitor alpha (IκBα) [47]. By facilitating the ubiquitination and subsequent degradation of IκBα, β-TRCP enables the release of NF-κB, allowing its translocation into the nucleus [47]. Once in the nucleus, NF-κB functions as a transcription factor, upregulating pro-inflammatory cytokines and initiating the inflammatory response [47]. The speckle-type BTB-POZ protein (SPOP), an adaptor for a Cullin 3-based ubiquitin ligase complex, serves as another key regulator. SPOP specifically recognizes the intermediate domain of MyD88 and targets it for proteasomal degradation. Loss of SPOP—either through knockdown or genetic ablation—leads to an abnormal accumulation of MyD88 protein. This positions SPOP as a negative regulator of the NF-κB pathway, suppressing IL-1β production in macrophages following LPS stimulation [48]. These interconnected pathways collectively drive the expression of pro-inflammatory cytokines, contributing to the cytokine storm characteristic of inflammatory diseases, including SIMD.

Nucleotide-binding oligomerization domain (NOD)-like receptors (NLRs) are critical proteins involved in immune response regulation and inflammation [49]. Among the NLR family, NOD1 and NOD2 are key receptors that recognize bacterial peptidoglycan components—γ-D-glutamyl-meso-DAP (iE-DAP) and muramyl dipeptide (MDP), respectively—and initiate signaling via the adaptor protein receptor-interacting serine–threonine kinase 2 (RIPK2) [50, 51]. Upon ligand binding, NOD1 and NOD2 recruit RIPK2 through caspase-activation-and-recruitment-domain (CARD)–CARD interactions, promoting K63-linked polyubiquitination of RIPK2 [51, 52]. This modification activates downstream signaling molecules, including TAK1 and IKK [53]. Multiple E3 ubiquitin ligases, such as TRAF6, cellular inhibitor of apoptosis 1 (cIAP1), cIAP2, and X-linked inhibitor of apoptosis (XIAP), regulate RIPK2 ubiquitination and the NOD1/2 signaling pathway [53]. Additionally, the CRL4DCAF8 E3 ligase plays a role in an LPS-induced SIMD mouse model by targeting nuclear receptor corepressor 1 (NcoR1) for ubiquitination and degradation [54]. Loss of NcoR1 disrupts its interaction with the transcription factor specificity protein 1 (SP1), leading to increased high mobility group box 1 (HMGB1) expression [54]. Elevated HMGB1 acts as an effector molecule, promoting pro-inflammatory cytokine production, exacerbating inflammation, and contributing to the development of SIMD [54]. Furthermore, the constitutive photomorphogenesis protein 1 homolog (COP1) is reported to ubiquitinate CCAAT/enhancer-binding protein beta (CEBPB), mitigating myocardial injury, reducing inflammatory responses, and alleviating SIMD [12].

### Ubiquitination and mitochondrial dysfunction

**Figure 5.**
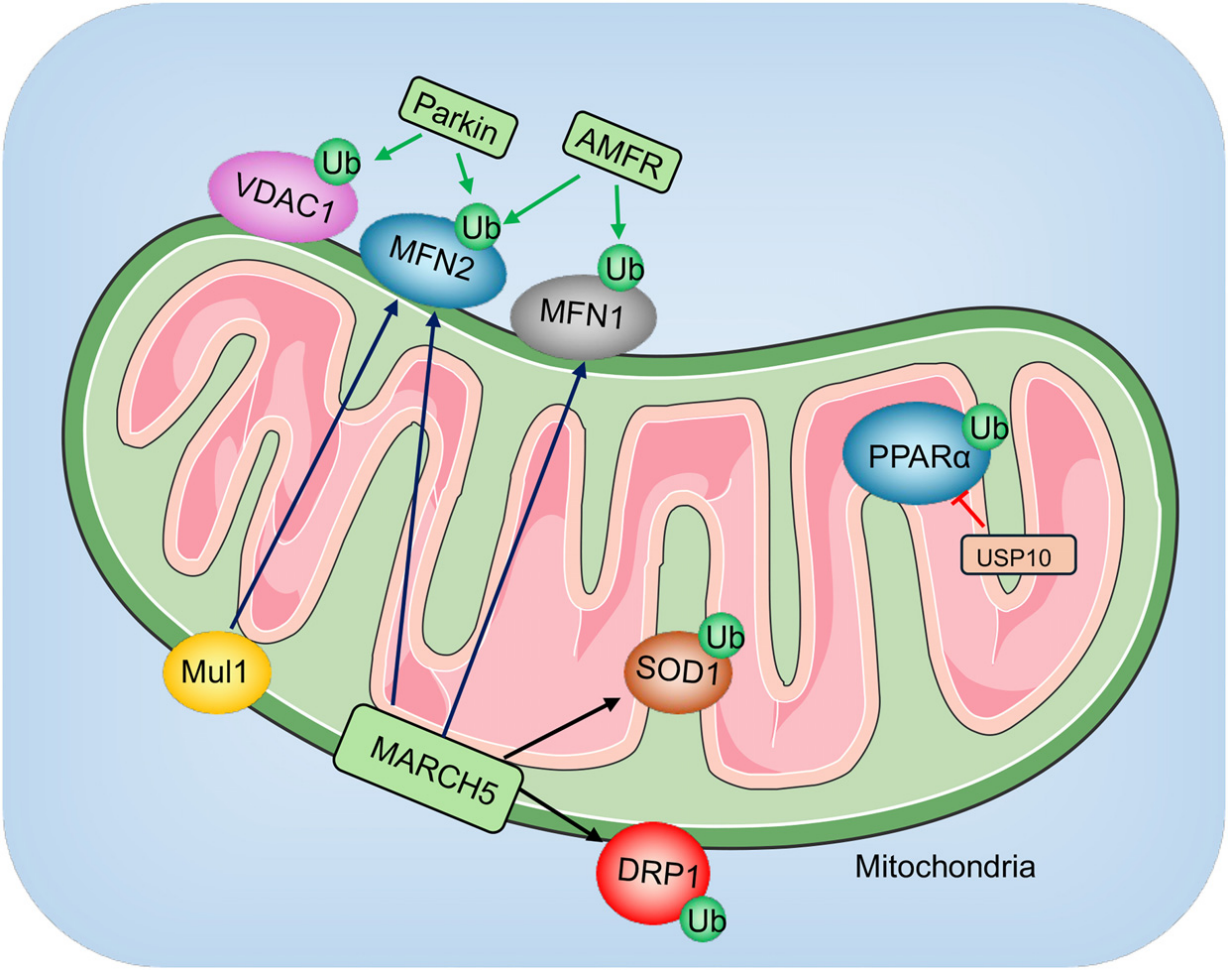
**Involvement of E3 ligases and DUBs inmitochondrial dysfunction.** Various E3 ligases and DUBs are involved in modulating mitochondrial function. Parkin ubiquitinates mitochondrial outer membrane proteins, such as VDAC1 and MFN2, promoting mitophagy. AMFR targets MFN1 and MFN2 for ubiquitination, while MARCH modifies MFN1, MFN2, DRP1, and SOD1. Mul1 specifically ubiquitinates MFN2. On the other hand, the DUB USP10 removes ubiquitin from PPARα, contributing to mitochondrial regulation. These post-translational modifications play critical roles in the progression of mitochondrial dysfunction. DUB: Deubiquitinating enzyme; USP10: Ubiquitin-specific protease 10; MFN2: Mitofusin-2; MFN1: Mitofusin-1; DRP1: Dynamin-related protein 1; SOD1: Superoxide dismutase 1; AMFR: Autocrine motility factor receptor; VDAC1: Voltage-dependent anion channel 1; MARCH: Membrane-associated RING-CH finger protein.

Mitochondrial function is tightly regulated by ubiquitination, which governs mitochondrial dynamics, quality control, and bioenergetics [55]. The UPS removes damaged mitochondrial proteins, preventing dysfunction [55]. However, during sepsis, excessive ubiquitination impairs mitochondrial function, leading to bioenergetic failure and increased ROS production [56]. This creates a vicious cycle where mitochondrial dysfunction amplifies ROS generation, causing further protein damage and proteasome overload [55, 56]. Moreover, reduced ATP levels resulting from mitochondrial impairment further suppress ubiquitination and proteasome activity, exacerbating the dysfunction [55, 56]. Notably, approximately 62% of the mitochondrial proteome may be ubiquitinated, emphasizing the critical role of ubiquitination in maintaining mitochondrial proteostasis [57]. Although direct evidence linking specific E3 ubiquitin ligases to SIMD remains limited, several E3 ligases known to regulate mitochondrial function in other pathological contexts may play roles in SIMD (Figure 5). For instance, Parkin, a key E3 ubiquitin ligase involved in mitochondrial quality control, functions within the PTEN-induced kinase 1 (PINK1)–Parkin pathway. Parkin ubiquitinates damaged mitochondrial outer membrane proteins, such as voltage-dependent anion channel 1 (VDAC1) and mitofusin-2 (MFN2), to facilitate mitophagy—the selective removal of dysfunctional mitochondria [58, 59]. Impaired Parkin activity has been associated with defective mitophagy, leading to the accumulation of damaged mitochondria and increased oxidative stress [58, 59]. While these mechanisms are well studied in neurodegenerative diseases, they may also play a significant role in the pathology of SIMD.

Autocrine motility factor receptor (AMFR), also known as Gp78, an E3 ubiquitin ligase associated with endoplasmic reticulum-associated degradation (ERAD), regulates mitochondrial dynamics by targeting MFN1 and MFN2 for degradation [60, 61]. Phosphorylation of AMFR at Ser538, mediated by p38 MAPK, reduces its ability to degrade MFN1 and MFN2, thereby influencing mitochondria-ER contact sites and mitochondrial dynamics [61]. Membrane-associated RING-CH finger protein 5 (MARCH5), localized to the mitochondrial outer membrane, is another critical regulator of mitochondrial dynamics. It ubiquitinates key fission and fusion proteins, including dynamin-related protein 1 (DRP1) and MFN1/2, to modulate mitochondrial morphology [62, 63]. Dysregulation of MARCH5 activity has been linked to excessive mitochondrial fission, fragmentation, and apoptosis [62, 63]. Additionally, MARCH5 plays a role in degrading misfolded superoxide dismutase 1 (SOD1), underscoring its involvement in mitochondrial protein quality control [64]. F-box and leucine-rich repeat protein 4 (FBXL4), a member of the SCF (SKP1-CUL1-F-box) E3 ligase family, helps maintain mitochondrial function by regulating components of the mitochondrial respiratory chain and oxidative phosphorylation [65]. Dysregulation of FBXL4 impairs ATP production [65], a hallmark of SIMD pathophysiology. Mitochondrial ubiquitin ligase 1 (Mul1), also known as MULAN, contributes to mitochondrial quality control by ubiquitinating MFN2 and promoting its degradation [66]. Aberrant Mul1 activity during sepsis disrupts mitochondrial fusion and worsens mitochondrial dysfunction [66]. In summary, although most studies on these E3 ubiquitin ligases have focused on their roles in other diseases, their well-established functions in mitochondrial regulation suggest they may also play a significant role in SIMD. Further research is necessary to confirm their specific contributions to SIMD pathogenesis and explore their potential as therapeutic targets.

### Ubiquitination and apoptotic signaling

Apoptosis is a hallmark of SIMD, with ubiquitination playing a critical role in regulating key components of apoptotic pathways [67]. E3 ubiquitin ligases, including MDM2, cIAP1/2, TRAF6, Parkin, Mul1, TRIM proteins, and XIAP, are central to apoptosis regulation (Figure 3) [68]. However, only a few of these ligases have been directly linked to the pathogenesis of SIMD. Among these, TRIM31, a member of the TRIM protein family, is notably upregulated in septic patients [69]. Studies show that reducing TRIM31 expression alleviates LPS-induced apoptosis, while its overexpression exacerbates it [69]. Mechanistically, TRIM31 interacts with and ubiquitinates TAK1, activating the TAK1-NF-κB signaling pathway. This activation drives both inflammatory responses and apoptosis, ultimately contributing to myocardial damage in septic conditions [69]. In addition, the cellular inhibitors of apoptosis proteins, cIAP1 and cIAP2, play a key role in regulating TNF receptor-mediated apoptosis by ubiquitinating receptor-interacting protein 1 (RIP1) [70]. This ubiquitination process prevents the formation of the necrosome complex, thereby maintaining a balance between apoptotic and necroptotic pathways. In sepsis, dysregulated cIAP1/2 activity worsens TNF-α-induced cell death, amplifying myocardial injury. Elevated cIAP1 levels are a characteristic feature in septic patients, and its knockdown in cardiomyocytes has been shown to reduce proliferation, increase apoptosis under LPS stimulation, and heighten inflammatory responses. These findings further underscore the pathological significance of cIAP1/2 in SIMD [70].

LPS exposure enhances TRAF6-mediated ubiquitination of Akt2, which correlates with increased expression of pro-apoptotic factors, including caspase-3 and caspase-12 [71]. Deletion of Akt2 alleviates LPS-induced cardiac dysfunction and apoptosis, indicating that TRAF6-driven ubiquitination of Akt2 facilitates apoptotic signaling in SIMD [11]. Furthermore, Parkin is upregulated in septic models following LPS treatment. This upregulation is associated with elevated levels of PINK1 and Beclin-1, leading to excessive mitophagy and apoptosis [71]. Although the specific substrates of Parkin in this context remain unidentified, its dysregulation underscores its critical role in mitochondrial dysfunction and subsequent myocardial damage in SIMD.

### Ubiquitination and calcium handling

Impaired calcium handling is a key feature of SIMD, with ubiquitination playing a pivotal role in regulating the stability and activity of calcium-regulatory proteins. Among these, SERCA2a is essential for maintaining calcium cycling and cardiac contractility. Dysregulation of SERCA2a and RyR2, often mediated by ubiquitination, contributes to calcium imbalance and cardiac dysfunction in SIMD (Figure 4) [72, 73]. SERCA2a expression and activity are frequently diminished during myocardial dysfunction [72]. Hydrogen sulfide (H_2_S) has been shown to regulate SERCA2a ubiquitination via S-sulfhydration of the E3 ubiquitin ligase muscle RING finger-1 (MuRF1) [72]. This modification stabilizes SERCA2a and influences cardiac contractility, underscoring ubiquitination as a key mechanism in SERCA2a regulation [72]. Recent studies have also identified methyltransferase METTL13 as a critical modulator of SERCA2a stability [74]. METTL13 promotes lysine methylation of Casitas B-lineage lymphoma (CBL), an E3 ubiquitin ligase, which inhibits CBL-mediated ubiquitination and subsequent degradation of SERCA2a [74]. In myocardial infarction (MI) models, METTL13 overexpression restores calcium transients and SERCA2a levels, whereas METTL13 knockdown exacerbates cardiac contractile dysfunction. Notably, silencing CBL mitigates the adverse effects of METTL13 deficiency, highlighting the regulatory role of the METTL13/CBL/SERCA2a axis in calcium handling and cardiac function [74]. Targeting ubiquitination pathways involved in SERCA2a regulation offers promising therapeutic strategies for treating SIMD. For example, enhancing the expression of small ubiquitin-like modifier type 1 (SUMO-1) has been shown to restore SERCA2a activity and improve cardiac function in heart failure models [75].

Recent studies indicate that RyR2 dysfunction, driven by ubiquitination and other proteolytic systems, contributes to myocardial dysfunction and related conditions [73]. In ischemia/reperfusion models, RyR2 protein levels significantly decline without corresponding changes in mRNA expression [73]. This reduction is mediated by both proteasomal and calpain-dependent degradation pathways [73]. Notably, in neonatal rat cardiomyocytes, inhibitors of either calpains or the proteasome prevent RyR2 loss following ischemia/reperfusion. This suggests that calpain activation, likely triggered by proteasomal degradation of calpastatin, initiates RyR2 breakdown, which is then exacerbated by proteasomal activity [73]. Such degradation disrupts calcium handling and reduces cardiac contractility. Additionally, the E3 ubiquitin ligase ubiquitin-conjugating enzyme E2 C (UBE2C) has been shown to ubiquitinate and degrade RyR2 in breast cancer cells, where it inhibits the Wnt/β-catenin signaling pathway [76]. While UBE2C’s direct involvement in SIMD remains unclear, its role in RyR2 regulation highlights the potential of ubiquitin-mediated degradation in modulating calcium dynamics in cardiomyocytes during stress conditions like sepsis.

## Deubiquitination in SIMD

Deubiquitination, the process of removing ubiquitin moieties from substrate proteins, is a tightly regulated mechanism mediated by DUBs [7, 13]. These enzymes play a crucial role in maintaining cellular homeostasis by reversing ubiquitination, which regulates protein stability, localization, and activity [7, 13]. Like E3 ligases, dysregulated DUB activity is strongly associated with pathophysiological processes, including inflammation, mitochondrial dysfunction, apoptosis, and calcium handling, all of which contribute to the onset and progression of SIMD (Table 2) [7, 13].

### Deubiquitination and inflammatory signaling

The process of deubiquitination plays a critical role in maintaining the balance and functionality of signaling molecules within TLR signaling pathways, ensuring proper immune responses while preventing dysregulation [13]. Various DUBs have been identified to perform specialized regulatory functions in these pathways (Figure 2). Among them, A20 (also known as TNF alpha-induced protein 3 [TNFAIP3]) has been extensively studied. Acting as a key inhibitor of TLR signaling, A20 removes K63-linked polyubiquitin chains from TRAF6, thereby terminating downstream signaling activity [77]. Interestingly, A20’s regulatory capacity extends beyond deubiquitination. It also possesses E3 ubiquitin ligase activity, enabling it to add K48-linked polyubiquitin chains to substrates such as RIPK1, marking them for proteasomal degradation [78]. Additionally, A20’s zinc-finger domains allow it to bind ubiquitinated proteins like IKKγ, effectively halting NF-κB activation by inhibiting upstream kinases [79]. Another notable DUB is cylindromatosis (CYLD), which functions as a negative regulator of NF-κB signaling by cleaving K63- and M1-linked polyubiquitin chains from signaling mediators, such as RIPK1 and IKKγ [80, 81]. Similarly, other DUBs regulate TLR signaling through unique mechanisms. For example, USP19 suppresses TLR3/4-mediated responses by interacting with TRIF and removing K27-linked polyubiquitin chains, thereby impairing TRIF recruitment [82]. Meanwhile, OTUD4, when phosphorylated, exhibits K63-specific activity and targets MyD88, effectively downregulating NF-κB signaling [83].

TRAF6 and TRAF3, key players in TLR signaling pathways, are tightly regulated by several DUBs. Myb-like, SWIRM, and MPN domains 1 (MYSM1) deubiquitinates both TRAF3 and TRAF6, thereby terminating antiviral signaling [84]. USP4, a potent inhibitor of TLR/IL-1R signaling, removes polyubiquitin chains from TRAF6, reducing the activation of NF-κB and AP-1 [85]. Similarly, UCHL1 targets TRAF3, limiting the production of proinflammatory cytokines [86]. Other DUBs, such as USP10, influence inflammatory responses by modulating the ubiquitination of TRAF6 and IKKγ, while monocyte chemotactic protein-induced protein 1 (MCPIP1) employs dual mechanisms—its DUB activity and RNase function—to suppress inflammation and degrade proinflammatory mRNAs like IL-6 and IL-1β [87]. Additionally, DUBs like USP25 and OTU deubiquitinase 7B (OTUD7B) play crucial roles in maintaining the stability of signaling components. USP25 prevents cIAP2-mediated degradation of TRAF3 [88], thereby enhancing cellular resistance to endotoxin shock. Meanwhile, OTUD7B stabilizes TRAF3 during noncanonical NF-κB signaling, preventing aberrant pathway activation [89]. While significant progress has been made in characterizing these DUBs, their specific roles in pathogen-specific contexts and immune defense mechanisms remain to be fully elucidated.

### Deubiquitination and mitochondrial dysfunction

Growing evidence underscores the pivotal role of dysregulated deubiquitination in mitochondrial dysfunction (Figure 5). USP30, a mitochondrial DUB localized on the outer mitochondrial membrane, plays a key role in regulating mitophagy [90]. By removing ubiquitin chains from Parkin substrates, USP30 counteracts Parkin-mediated ubiquitination, effectively acting as a brake on mitophagy [90]. Dysregulation of USP30 impairs the clearance of damaged mitochondria, exacerbates mitochondrial dysfunction, and contributes to oxidative stress and myocardial injury [90]. In myocardial cell senescence models, USP30 overexpression suppresses mitophagy, promotes ROS production, and accelerates cellular aging by reducing Beclin1 and LC3II levels while increasing the expression of p53, p21, and p16 [91]. In contrast, USP28 overexpression has been shown to alleviate cardiac remodeling, dysfunction, lipid accumulation, and mitochondrial impairment in mouse models of type 2 diabetes induced by a high-fat diet and streptozotocin [92]. Mechanistically, USP28 uses its ubiquitin carboxyl-terminal hydrolase (UCH) domain to bind to the DNA-binding domain (DBD) of PPARα [92]. This interaction facilitates lysine-specific deubiquitination, stabilizing PPARα and upregulating Mfn2 expression [92]. The resulting enhancement in mitochondrial integrity and function helps prevent cardiac dysfunction associated with diabetic cardiomyopathy [92].

**Table 2:** DUBs involved in various biological processes in the pathogenesis of SIMD

**DUBs**	**Substrates**	**Biological processes**	**References**
A20	TRAF6 RIPK1	Inflammation	[77, 78]
CYLD	RIPK1 IKKγ	Inflammation	[80, 81]
USP19	TRIF	Inflammation	[82]
OTUD4	MyD88	Inflammation	[83]
MYSM1	TRAF3 TRAF6	Inflammation	[84]
USP4	TRAF6	Inflammation	[85]
UCHL1	TRAF3	Inflammation	[86]
USP10	TRAF6 IKKγ	Inflammation	[87]
USP25	TRAF3	Inflammation	[88]
OTUD7B	TRAF3	Inflammation	[89]
USP28	PPARα	Mitochondrial dysfunction	[92]
A20	TRAF6 RIPK1	Mitochondrial dysfunction	[93, 94]
USP7 USP10 USP24	p53	Apoptosis	[97]
USP7	SOX9	Apoptosis	[100]
USP25	SERCA2a	Calcium handling	[102]
JOSD2	SERCA2a	Calcium handling	[103]
OTUD1	PDE5A	Calcium handling	[104]
USP5	Cav3.2	Calcium handling	[105]

SIMD: Sepsis-induced myocardial dysfunction; DUB: Deubiquitinating enzyme; SERCA2a: Sarcoplasmic/endoplasmic reticulum calcium ATPase 2a; TRAF6: TNF receptor-associated factor 6; IKKγ: Inhibitor of NF-κB kinase subunit gamma; RIPK1: Receptor-interacting serine–threonine kinase 1; MYD88: Myeloid differentiation primary response 88; USP19: Ubiquitin-specific protease 19; USP4: Ubiquitin-specific protease 4; USP10: Ubiquitin-specific protease 10; USP28: Ubiquitin-specific protease 28; USP7: Ubiquitin-specific protease 7; USP24: Ubiquitin-specific protease 24; USP5: Ubiquitin-specific protease 5; USP25: Ubiquitin-specific protease 25; TRAF3: TNF receptor-associated factor 3; MYSM1: Myb-like, SWIRM, and MPN domains 1; JOSD2: Josephin domain-containing 2; PDE5A: Phosphodiesterase 5A.

In addition to their impact on mitochondrial dynamics, DUBs also influence inflammatory pathways that intersect with mitochondrial health. A20, for example, mitigates NF-κB signaling by removing K63-linked ubiquitin chains from intermediates, such as RIPK1 and TRAF6 [93, 94]. Similarly, USP14 highlights the link between deubiquitination and mitochondrial regulation [95]. This DUB modulates cellular proteostasis and indirectly affects mitochondrial function by regulating ROS production and bioenergetics [95]. Experimental evidence indicates that USP14 inhibition enhances mitochondrial turnover, reduces oxidative damage, and protects against myocardial injury in sepsis models [96]. Collectively, these findings underscore the multifaceted roles of DUBs like USP30, A20, and USP14 in regulating mitochondrial function and inflammation (Figure 5), suggesting their potential involvement in the pathogenesis of SIMD.

### Deubiquitination and apoptotic pathways

A variety of DUBs mediate the deubiquitination of pro-apoptotic proteins, such as p53, Bax, and Bim [97]. For instance, p53 is deubiquitinated by USP7, USP10, and USP24, which stabilizes p53 and promotes p53-dependent cell growth suppression and apoptosis [97]. Similarly, USP12 targets Bax [98], while USP27x acts on Bim [99] (Figure 3). However, whether these DUBs directly regulate p53, Bax, and Bim in the pathogenesis of SIMD remains unclear. Several DUBs, including USP7 and OTUD1, have been implicated in myocardial injury. In a murine model of sepsis established via cecal ligation and puncture (CLP), USP7 was shown to enhance SOX9 expression through deubiquitination [100]. SOX9, in turn, suppressed miR-96-5p expression by binding to its promoter region, which led to increased NLRP3 expression and exacerbated sepsis-induced myocardial injury and cardiomyocyte death [100]. Meanwhile, OTUD1 plays a critical role in regulating inflammatory responses, innate immunity, oxidative stress, and ROS-mediated cell death pathways [101]. In Otud1−/− mice, sepsis models demonstrated heightened inflammation, oxidative damage, and cell death. Furthermore, OTUD1 was found to negatively regulate canonical NF-κB activation, apoptosis, and necroptosis [101].

### Deubiquitination and calcium handling

Deubiquitination plays a critical role in maintaining calcium handling in cardiomyocytes, a process essential for preserving cardiac contractility and preventing dysfunction in SIMD [7]. The delicate balance between ubiquitination and deubiquitination regulates the stability and function of calcium-handling proteins [7], emphasizing the importance of DUBs in SIMD pathogenesis. USP25 directly interacts with SERCA2a, removing K48-linked ubiquitin chains to deubiquitinate the protein (Figure 4) [102]. This action prevents proteasomal degradation of SERCA2a, stabilizing its levels and supporting calcium cycling in cardiomyocytes [102]. Notably, USP25 deficiency exacerbates cardiac dysfunction and hypertrophy under pathological conditions, whereas restoring its expression alleviates these effects [102]. Similarly, Josephin domain-containing 2 (JOSD2), another deubiquitinase, stabilizes SERCA2a, enhancing its activity and maintaining calcium homeostasis (Figure 4) [103]. JOSD2 is upregulated in hypertrophic myocardium, and its overexpression prevents angiotensin II-induced cardiac hypertrophy. In contrast, JOSD2 deficiency disrupts calcium regulation, resulting in cardiomyocyte hypertrophy [103]. These findings further underscore JOSD2’s critical role in preserving calcium signaling and cardiac function [103]. OTUD1 also influences calcium handling by stabilizing phosphodiesterase 5A (PDE5A), a key regulator of the cGMP-PKG-SERCA2a signaling axis [104]. By deubiquitinating PDE5A, OTUD1 prevents its degradation. However, this stabilization disrupts calcium signaling and exacerbates myocardial injury under stress conditions [104]. Experimental models reveal that OTUD1 knockdown mitigates cardiac dysfunction induced by isoprenaline (ISO) or MI, partly by preserving SERCA2a function and calcium cycling. These results highlight OTUD1’s detrimental role in calcium regulation during myocardial stress [104].

USP5 modulates calcium signaling by affecting T-type calcium channels (Cav3.2) and VGCCs. Specifically, USP5 stabilizes Cav3.2 expression on the cell surface by deubiquitinating the channel, thereby regulating calcium influx [105]. Although its role in SIMD remains unexplored, USP5’s involvement in calcium regulation suggests it may influence SIMD pathogenesis. Similarly, USP2-45 has been implicated in VGCC regulation by interacting with the CaVα2δ-1 subunit [106]. This interaction promotes the deubiquitination of CaV1.2 and its ancillary subunit, thereby altering channel stability and function [106]. While these findings emphasize USP2-45’s role in calcium signaling, its specific contribution to SIMD is yet to be determined. Collectively, DUBs, such as USP25, JOSD2, OTUD1, and USP5, play critical roles in regulating calcium-handling proteins and maintaining cardiomyocyte function under stress (Figure 4). Exploring these mechanisms offers valuable insights into SIMD pathogenesis and reveals potential therapeutic targets.

## Conclusion

The ubiquitination and deubiquitination systems are pivotal regulatory mechanisms in the progression of SIMD. These PTMs control essential biological processes, such as inflammation, mitochondrial function, apoptosis, and calcium handling, all of which are integral to SIMD pathogenesis [3–8]. Dysregulated ubiquitination, driven by E3 ligases, often leads to the proteasomal degradation of key regulatory proteins, amplifying inflammation, oxidative stress, and programmed cell death [3–8]. Concurrently, imbalances in DUBs disrupt protein homeostasis, further exacerbating myocardial injury [3–8]. The intricate interplay between these systems and other modifications, such as phosphorylation, compounds the complexity of the molecular pathways involved in SIMD [3–8]. A diverse array of E3 ligases and DUBs regulate the biological processes contributing to SIMD pathogenesis [3–8]. Under physiological conditions, these systems operate in a finely tuned balance to maintain protein stability and function. However, disruption of this balance can impair critical cellular processes, driving the onset and progression of SIMD [3–8]. For instance, TRAF6 is pivotal in inflammatory signaling [45, 46], while Parkin plays an essential role in mitochondrial quality control [58, 59]. Additionally, DUBs like USP25 and JOSD2 stabilize SERCA2a to preserve calcium homeostasis [102, 103], while USP30 counteracts Parkin-mediated ubiquitination to regulate mitophagy [90, 91]. These mechanisms underscore the importance of ubiquitination and deubiquitination in maintaining cardiac function. Despite these insights, many findings are extrapolated from broader studies on cardiac or inflammatory diseases, leaving critical gaps in understanding their precise roles in the unique context of SIMD. Some E3 ligases and DUBs modify multiple substrates, adding further complexity to their regulatory networks. For example, MARCH ubiquitinates MFN1, MFN2, SOD1, and DRP1 [62–64], while DUBs like A20, USP4, USP10, and MYSM1 target DCAF6 [84, 85, 87, 94]. Key questions remain unresolved, such as which E3 ligases or DUBs predominantly regulate specific substrates in SIMD, whether these modifications occur simultaneously, and the extent of their cell-specific effects. Addressing these gaps will require more precise investigations to unravel the roles of E3 ligases and DUBs in SIMD pathogenesis. A comprehensive understanding of these mechanisms will lay the groundwork for developing targeted therapeutic strategies to mitigate SIMD progression.

One major challenge in studying SIMD is the limited direct evidence linking specific E3 ligases and DUBs to its pathogenesis. The molecular crosstalk between these systems and other pathways—such as mitochondrial dynamics and calcium signaling—remains poorly understood. Furthermore, the lack of advanced tools and models to study these systems *in vivo* under septic conditions presents a significant barrier to validating preclinical findings. Addressing these challenges requires a multifaceted approach. Future research should prioritize characterizing the specific roles, substrates, and regulatory mechanisms of E3 ligases and DUBs in SIMD. This includes investigating their interactions with other PTMs, such as phosphorylation, as well as their involvement in interconnected pathways like mitochondrial dynamics, calcium signaling, and inflammation. High-resolution techniques, such as spatiotemporal imaging and high-throughput proteomics, will be essential for unraveling these intricate molecular networks. Additionally, the development of physiologically relevant animal models and organoid systems that closely replicate the complex environment of sepsis is critical. Such models will enable the study of cell- and tissue-specific effects and the temporal dynamics of ubiquitination-related processes under septic conditions. Translational and clinical studies are equally important for bridging the gap between basic research and patient care. Developing specific inhibitors or activators targeting these enzymes could pave the way for novel therapeutic interventions. Leveraging cutting-edge technologies, such as CRISPR-based functional screening, single-cell sequencing, and advanced proteomics will provide deeper insights into the ubiquitination–deubiquitination landscape in SIMD. Ultimately, these efforts will enhance our understanding of SIMD pathogenesis, facilitate the development of targeted therapies to restore balance within the UPS, and mitigate myocardial injury. By addressing these critical gaps, future research has the potential to significantly improve outcomes for patients suffering from SIMD.
